# Rectifying Resistive Memory Devices as Dynamic Complementary Artificial Synapses

**DOI:** 10.3389/fnins.2018.00755

**Published:** 2018-10-22

**Authors:** Dan Berco

**Affiliations:** School of Electrical and Electronic Engineering, Nanyang Technological University, Singapore, Singapore

**Keywords:** artificial neural networks, brain inspired computing, dynamic artificial synapses, memristors, rectifying synapses

## Abstract

Brain inspired computing is a pioneering computational method gaining momentum in recent years. Within this scheme, artificial neural networks are implemented using two main approaches: software algorithms and designated hardware architectures. However, while software implementations show remarkable results (at high-energy costs), hardware based ones, specifically resistive random access memory (RRAM) arrays that consume little power and hold a potential for enormous densities, are somewhat lagging. One of the reasons may be related to the limited excitatory operation mode of RRAMs in these arrays as adjustable passive elements. An interesting type of RRAM was demonstrated recently for having alternating (dynamic switching) current rectification properties that may be used for complementary operation much like CMOS transistors. Such artificial synaptic devices may be switched dynamically between excitatory and inhibitory modes to allow doubling of the array density and significantly reducing the peripheral circuit complexity.

## Introduction

Ever since the scientific community’s revival of interest in memristors (Chua, 1971; Chua and Kang, 1976) was triggered by publications in the last decade such as Strukov et al. (2008), these devices have been extensively used for the implementation of artificial neural networks (ANN) in brain-inspired computational platforms. Within this domain, crossbar array architectures are promising candidates for achieving high-densities (∼10^15^ bits/cm^2^) similar to the human cerebral cortex (∼10^14^ synapses), when configured in 3D stacking (Kügelera et al., 2009), due to the nanoscale device dimensions (Aratani et al., 2007; Jo et al., 2010). In addition, crossbar arrays are very efficient, in terms of calculation time and energy expenditure, when performing matrix-vector dot product operations, that form the basis for machine learning algorithms (Hu et al., 2012). Several demonstrations of resistive random access memory (RRAM) array implementations proved to be very successful in tasks such as image classification (Prezioso et al., 2015). The sizes of these networks range from small scales of only few neurons (Kim et al., 2012; Prezioso et al., 2015), medium levels (Park et al., 2015; Hu et al., 2016) and up to larger scale that incorporate hundreds of neurons (Yao et al., 2017) and even up to 10^5^ synaptic connections (Burr et al., 2015).

Resistive random access memory arrays are designed to imitate the functionality of biologic synaptic networks. A chemical synapse is a gapped connection between two neurons through which communication takes place (Pereda and Faber, 1996; Kandel et al., 2000; Nicholls et al., 2001). A typical neuron can have several thousands of synapses which mostly connect axons in a presynaptic neuron to dendrites in postsynaptic neuron. Inter-neural signaling occurs by the release of neurotransmitters from the presynaptic neuron into the gap (i.e., synaptic cleft) that in turn is collected by receptors in the postsynaptic neuron. The molecular neurotransmitters are kept in sacs called synaptic vesicles. During signaling, these vesicles are released into the synaptic cleft and bind to receptors on the postsynaptic neuron. Once the signal is delivered, the transmitters are evacuated from the receptors through potential mechanisms such as enzymatic degradation, or absorbed back into the presynaptic neuron by specific transporters. The postsynaptic potential response is classified as being either excitatory or inhibitory and determined by the type of neurotransmitter (Glutamate or γ-aminobutyric acid) (Nakanishi, 1992). Two key characteristics resulting from this behavior are the so called long-term potentiation (LTP) and long-term depression (LTD) and the synaptic weight (connection strength) is modulated by this neural activity. A recent study showed that actually both types of neurotransmitter could be released simultaneously during synaptic activity (Root et al., 2014). Moreover, neurotransmitters have been shown to be able to actually exchange roles during early stages of brain development (Ben-Ari, 2002).

Biologic neural networks have evolved over hundreds of millions of years to easily and efficiently perform tasks that state of the art computers find difficult. The operation of a man-made ANN should thus be true to the source as much as possible with respect to a building block artificial device. In order to achieve this target, artificial synaptic devices (ASDs) should imitate as much as possible the traits that biological synapses have. Although current understanding of neural networks and synapses is nowhere near complete, one may assume that such ASDs would be the best option for future ANN implementations. Some of these basic features include having a large dynamic range and multilevel operation to match the analog nature of the biological synaptic weight changes. These traits translate to higher accuracies, more degrees of freedom for weight adjustment and robustness during network training. A positive correlation exists between device dimensions and the number of states it can support (e.g., multilevel resistance) (Kuzum et al., 2013). However, sizing up the device will increases the overall current and power consumption as well as reduce the potential density.

The potential ability to implement symmetric weight changes may play a role in the simplification of an ANN peripheral control system. Biologic neural networks are very adaptive and can easily compensate for asymmetric weight changes especially when hundreds of neurons are involved in determining the weight of a synaptic junction. State machine based control systems on the other hand, are best suited to operate with well known and predictable parameters. In order to deal with asymmetric or random parametric distributions, an elaborate feedback system must be implemented and incorporated into the controller. In this sense, an ASD having a potential for incrementally small changes in both the up and down directions may simplify the controller design. A desirable corresponding weight parameter (e.g., conductance in RRAM) should thus be both symmetric with regards to the direction of change (increase or decrease) and differentially linear in magnitude of step change. Nonetheless, RRAMs (being a promising candidate in terms of low-power and high-density) (Wong et al., 2012) and other types of memristors show non-linear conductance as well as asymmetric conductivity changes (Lee et al., 2010) in response to successive set and reset pulses (Alibart et al., 2012; Chen et al., 2015; Wang et al., 2016) that complicate the task of designing a control state machine and sensing circuitry. This in turn affects both the potentially achievable network accuracy and overall performance.

An even more critical attribute expected from an ideal ASD would be the ability to reconfigure dynamically during real time operation between the excitatory and inhibitory response modes in a similar manner to a biologic synapse. Current RRAM devices are not able to reproduce this feature since they are passive devices by nature (once formed) and intentionally operated to comply with the linearity requirements as much as possible. It is virtually impossible for an RRAM to display this dynamic attribute without adding an additional control terminal to modulate the material properties by the field effect. In other words, it should have a bipolar conductance that is distinct characteristic of active devices. Adding a control terminal would damage the linearity and seriously downgrade the high-integration capabilities.

A common architectural solution is used to work around the dynamic reconfiguration issue by employing a differential approach. Instead of representing a synaptic gap by a single RRAM, two devices are used in a differential manner (Bichler et al., 2012; Prezioso et al., 2015). In this way, the synaptic weight-current is evaluated through a differential amplifier to determine whether the synaptic gap represents an excitatory or an inhibitory state. Needless to say, the prospective array density is reduced to half in addition to the added complexity required from the control and sensory circuitry. Recent publications have demonstrated ASDs with dynamic capabilities (mimicking either LTP or LTD behaviors as a function of a modulation bias) through the use of FET structures (Kim et al., 2013; Tian et al., 2015, 2016, 2017; Yang et al., 2015). As mentioned previously, these devices rely on at least one additional modulation terminal and an associated bias voltage to control the conductance polarity through the field effect. However, they are operated using very large biases (tens of volts) that seriously compromise their integration possibility with modern CMOS architectures. Moreover, the physical dimensions of these devices are very large (tens of micrometers), in addition to having a lateral structure (as opposed to vertical stacking) that is not optimal for high-density 3D integration. State of the art ASDs are thus still far from being able to truly reproduce the behavior of biologic synapses.

Another type of biologic synapses is based on fast conductive links between neurons capable of transmitting and receiving electrical signals (Pereda and Faber, 1996; Kandel et al., 2000). These links contain numerous ion channels (i.e., connexons) scattered along plasma membranes to form the connection between the pre- and post-synaptic neurons (Connors and Long, 2004; Michael et al., 2004). Some of these junctions demonstrate a rectifying behavior while being stimulated by electric pulses resulting in a preferred direction for ion flow (Hormuzdi et al., 2004; Landisman and Connors, 2005; Haas et al., 2011). In this light, another subclass of micrometer sized RRAMs was shown to have a unidirectional current rectification property along with non-volatile, multilevel resistive states (Yoon et al., 2015; Kim et al., 2016). In addition, Kim et al. (2018) demonstrated a rectifying micrometer ASD with transient (volatile) current-voltage dependence. Unfortunately, all these devices fall short in terms of both size and high-density integrability that play a key role in the implementation of ideal ANN.

Recently, Berco et al. (2018) presented a proof of concept for a nanoscale current rectifying dynamic RRAM. This ASD, of merely a few nanometers in size, dissipates only several picowatts of power during operation while having the ability to dynamically flip its current rectification direction thus effectively implementing both excitatory and inhibitory synaptic functionalities without the need for a modulation terminal. This operation mode allows for doubling the array size when compared to the common differential approach discussed previously (Bichler et al., 2012; Prezioso et al., 2015). Conductance-weight changes may be implemented by using a digital methodology (as opposed to the ubiquitous analog model). In this manner, a group of nanoscale ASDs are grouped together to represent a single artificial synapse where each member of the group plays the role of a rectifying connexon at the expense of layout resources. Setting the number of current-rectifying devices to a specific direction, being either positive rectification (PR) or negative rectification (NR), in relation to the others (being in the opposite direction), effectively determines the total conductance for each rectification direction. These artificial rectifying connexons (ARCs) may be individually toggled and the overall synaptic weight digitally manipulated in a similar manner to the LTP and LTD in biologic synapses.

## Artificial Synaptic Device Implementation

**FIGURE 1:**
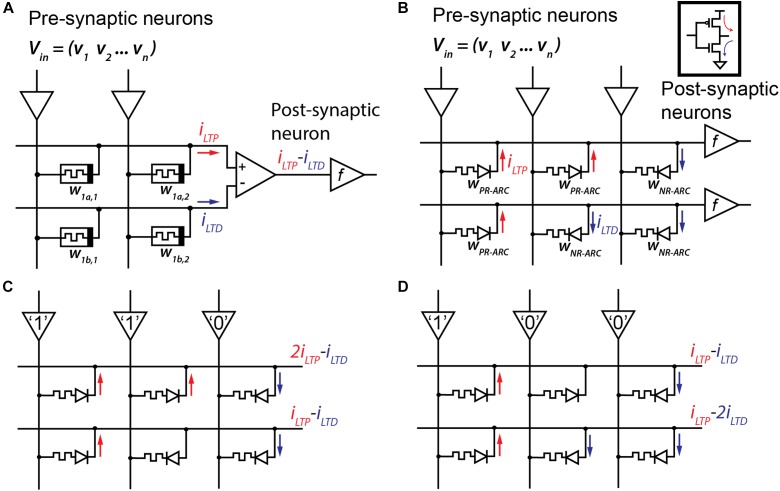
**(A)** A common approach for emulating LTP and LTD using a memristors crossbar array as an ANN weight matrix (Prezioso et al., 2015; Bichler et al., 2012). Two memristors (*W_*1a,1*_* and *W_*1b,1*_*) represent a single artificial synapse and their induced current, based on a pre-programmed conductance, is summed in a differential manner to determine the synaptic potentiation or depression. **(B)** An ARC-based implementation can both double the array density and simplify the peripheral circuitry by allowing dynamic switching of the rectification direction thus implementing either an excitatory or inhibitory weight parameter at each junction (much like a CMOS gate shown in the inset). **(C)** Sample response of an ARC-based ANN to a generic input (1,1,0). **(D)** A different input (1,0,0) would produce a different response from the same network setting.

Memristor arrays for hardware implementations of ANN are extremely efficient in performing matrix vector dot products as weighted-sum operations. A widely used RRAM-based differential array architecture is depicted in Figure 1A (Bichler et al., 2012; Prezioso et al., 2015). In this approach, two passive devices are used to determine a single synaptic weight in a differential manner thus allowing for both positive and negative parametric values. Programming the analog conductance levels may be done either during network training or on the fly to emulate the LTP and LTD synaptic behaviors. The sensing circuitry is based on a differential amplifier driving an activation function module (marked as *f*). However, analog RRAM operation (conductivity adjustment) (Lee et al., 2010; Alibart et al., 2012; Chen et al., 2015; Wang et al., 2016) usually requires complex pulsing schemes to account for its non-linear nature which complicates the design of a digital controller. An ARC-based implementation is given in Figure 1B (Berco et al., 2018). The operation principal allows for dynamically switching of the rectification direction resulting in either an excitatory or inhibitory weight parameter at each junction. In this manner, a single active device can be set to either push or pull current (much like the CMOS couple shown in the inset) effectively doubling the prospective density and simplifying the peripheral circuitry considerably.

A sample response of an ARC-based ANN to a generic input (1,1,0) is demonstrated in Figure 1C. In this example, some ARCs are configured as a PR-ARC (current direction marked by a red arrow) while the others are configured as an NR-ARC (current direction marked by a blue arrow). A high-voltage input “1” will result in current flowing into the output neuron through the PR-ARC. In the same manner, a low-voltage input “0” will result in current flowing out of the output neuron through the NR-ARC. ARCs that are configured in an opposite direction (in relation to the input value) will produce a zero current response. Figure 1D depicts the response of the exact same network configuration to a different input vector (1,0,0). In this manner, both network training and learning may be implemented.

**FIGURE 2:**
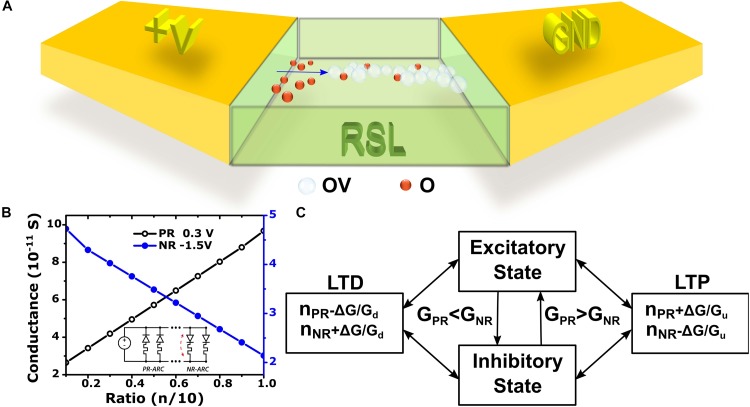
**(A)** Schematic diagram of OV-O distribution in the RSL of an ARC after forming where the gap in the CF (indicated by a blue arrow) yields a rectifying behavior. **(B)** A digital approach for synaptic weight adjustment demonstrated by Spice simulation (using a generic behavioral model for a group of 10 ARCs). The LTP conductance (black curve) depends on the ratio of PR-ARCs to NR-ARCs with a bias of 0.3 V and the LTD conductance (blue curve) depends on the ratio of NR-ARCs to PR-ARCs with a bias of –1.5 V while the inset shows the circuit under simulation where individual ARC are consecutively flipped from PR to NR and *vice versa*. **(C)** An abstract model for synaptic operation as proposed by Berco et al. (2018).

Figure 2A depicts a schematic diagram of a lateral structured RRAM device configured as an ARC for illustrative purposes. The device is comprised of a metal-oxide-based resistive switching layer (RSL) placed between two conductive electrodes. The forming of a conductive filament (CF, an aligned path of current conducting defects) is done using a specific current compliance limit that yields an uneven distribution of oxygen vacancies (OV) and oxygen species (O) (Berco and Tseng, 2016; Berco et al., 2018). The figure depicts positively charged OV being pushed away from the anode and accumulated near the cathode while the negatively charged O ions are drawn to the anode. The forming process is arrested by the current compliance setting before a continuous CF from anode to cathode is able to form. The resulting gap (indicated by a blue arrow) yields a current rectifying behavior (Berco et al., 2018).

The dynamic nature of ARCs may be utilized for real time modulation of the ASD junction plasticity, using a plurality of devices, by changing the ratio of the number of PR to NR-ARCs. A digital approach (contrary to the common analog treatment) (Lee et al., 2010; Alibart et al., 2012; Chen et al., 2015; Wang et al., 2016) using ARCs for implementing LTP and LTD synaptic weight adjustment is summarized in Figure 2B. Using this concept, a group of ARCs are treated as a single ASD (consuming more area). This implementation was verified with Spice simulations using a behavioral model based on the experimental data published by Berco et al. (2018). The circuit under simulation is composed of 10 ARCs connected in parallel to a single DC voltage source (Figure 2B inset) representing the input value (either “0” or “1”). The LTP simulation progresses by consecutive flipping an NR-ARC to a PR-ARC starting from *n* = 1 to *n* = 10 and calculating the overall conductance under positive bias of 0.3 V. The LTD simulation is done by consecutive flipping a PR-ARC to an NR-ARC in the same manner under a negative bias of -1.5 V. The conductance results in Figure 2B show a good linear behavior when depicted as a function of the ratio of PR-ARCs (*n*) to NR-ARCs (10-*n*) and *vice versa*. The positive slope for PR may thus be used to implement an excitatory synaptic weight change (increased current flow to the postsynaptic circuitry for a positive input vector) while the negative slope for NR may be used for an inhibitory synaptic weight (increased current flow from the postsynaptic circuitry for a negative input vector).

## Abstract Model

Figure 2C depicts a block diagram of an abstract model for synaptic operation based on *n* ARCs connected in parallel as proposed by Berco et al. (2018). The number of PR-ARCs in the group is marked as *n_*PR*_* and of NR-ARCs as *n_*NR*_*. *G_*PR*_* is the combined conductance of the PR-ARCs and *G_*NR*_* of the NR-ARCs. The conductivity of a single PR-ARC is *G_*u*_* and *G_*d*_* of a single NR-ARC. Both parameters may be modeled after experimental data by using a behavioral lookup table. The synaptic model transitions from an excitatory state to an inhibitory one once the positive conductivity surpasses the negative conductivity and *vice versa*. The synaptic weight change *ΔG* corresponds to spiking timing alignment in biologic synapses and is determined by network training and operation. In this manner, the number of ARCs which are flipped from a NR state to a PR one is determined by the ratio *ΔG/G_*u*_* for LTP. In a similar way, the number of ARCs which are flipped from a PR state to a NR one is determined by the ratio *ΔG/G_*d*_* for LTD.

## Logic Gate Implementation

**FIGURE 3:**
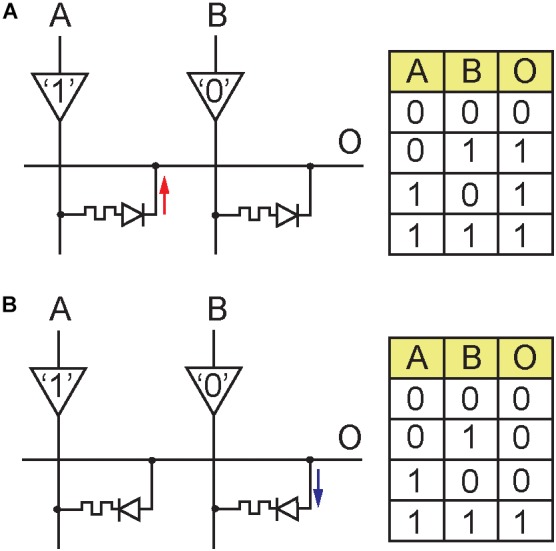
Implementation of logic gates with ARCs. The directionality of the ARC determines either a push or pull functionality and as a result the logic output value. **(A)** OR gate. **(B)** AND gate.

ARCs may also be used for the implementation of logic gates as depicted in Figure 3. Programming entire crossbar arrays could be utilized in this manner to implement in-memory computing schemes. Figure 3A shows the implementation of an OR gate. Both ARCs are programmed to rectify current from the inputs toward the output. A high logic level setting of any of the inputs would result in current flow and charging of the output parasitic capacitance to “1”. When both inputs are set low the output will either retain a low logic level or discharge through leakage to “0”. Figure 3B gives the implementation of an AND gate. In this case, both ARCs are set to rectify in an opposite direction (from output to inputs). Only when both inputs are set to a high level the output will charge through leakage to “1”. If any of the inputs is set to a low level, a discharge path will occur which will force the output to “0”.

## Conclusion

In summary, a nanoscale RRAM with dynamic current rectification properties may be used as an ASD in neural networks to effectively double the array density for some applications and significantly reduce the required complexity from the peripheral circuitry (both sensing and control). This device is analogous to a biologic connexon (gap connection between synapses) that, when aggregated in a group, define the overall synaptic directionality and weight with respect to ion motion. An ARC may be dynamically toggled between positive and negative rectifications states thus allowing for a complementary operation (much like CMOS devices) of artificial synapses (as opposed to the linear analog scheme common to passive RRAM-based networks). In addition, the synaptic weight may be controlled in a digital manner by using a plurality of devices grouped together by changing the ratio of the number of positive-rectifying to negative-rectifying ones. Furthermore, the LTP and LTD behaviors of biologic synapses may be emulated as well.

## Author Contributions

The author confirms being the sole contributor of this manuscript and has approved it for publication.

## Conflict of Interest Statement

The author declares that the research was conducted in the absence of any commercial or financial relationships that could be construed as a potential conflict of interest.
